# Beyond the cross‐section: Rethinking the intention–behaviour gap through a conceptual and methodological lens

**DOI:** 10.1111/bjhp.70046

**Published:** 2025-12-30

**Authors:** Darko Jekauc, Manuel C. Voelkle, Falko F. Sniehotta, Claudio R. Nigg

**Affiliations:** ^1^ Institute of Sports and Sports Science Karlsruhe Institute of Technology Karlsruhe Germany; ^2^ Institute of Psychology University of Freiburg Freiburg Germany; ^3^ Department of Public Health, Social and Preventive Medicine, Medical Faculty Mannheim Heidelberg University Mannheim Germany; ^4^ Department of Health Sciences, Institute of Sport Science University of Bern Bern Switzerland

**Keywords:** dynamic behaviour modelling, ecological momentary assessment, intention–behaviour gap, stability, within‐person variability

## Abstract

**Objectives:**

The intention–behaviour gap (IBG) remains a persistent challenge in health psychology. While intentions are widely recognized as proximal determinants of behaviour, they frequently fail to translate into action. This conceptual review aims to critically evaluate dominant models of the IBG and propose a dynamic, multidimensional reconceptualization of intention–behaviour processes.

**Methods:**

This conceptual review critically evaluates dominant models of the IBG–the Explained Variance Approach and the Action Control Framework. Special emphasis is placed on methodological constraints associated with between‐person research designs, which inadequately capture the temporal and contextual variability of behavioural enactment.

**Results:**

Theoretical and empirical limitations are identified in existing IBG models, including their failure to address intra‐individual fluctuations, situational contingencies and the time‐indexed nature of behavioural regulation. In response, we propose a framework emphasizing intensive longitudinal designs, within‐person assessment, ecological momentary methods and person‐specific modelling techniques to better capture intention–behaviour dynamics.

**Conclusions:**

To close the IBG, future research should integrate dynamic methodologies and real‐time interventions that align with situational and motivational states. We advocate for context‐sensitive strategies, such as just‐in‐time adaptive interventions and implementation intentions, to enhance behavioural enactment. This reconceptualization offers a pathway towards more precise theory and effective intervention in health behaviour change.

**Statement of Contribution**

**What is already known on this subject?**

- Research on the intention–behaviour gap (IBG) has predominantly used between‐person designs that overlook the dynamic, within‐person nature of behaviour regulation.
- Existing theories, such as the Theory of Planned Behaviour and the Health Action Process Approach, focus on motivational and volitional processes but treat intention as a relatively stable construct
- Traditional methodological approaches provide limited insight into how intentions fluctuate across time and context and how these fluctuations shape behaviour in everyday life.

**What does this study add?**

- This paper advances the multidimensional difference framework, which conceptualizes the IBG as a dynamic, within‐person discrepancy that varies across person, temporal, contextual and behavioural dimensions.
- It integrates conceptual and methodological reasoning by outlining how intensive longitudinal methods, micro‐randomized trials and person‐specific modelling can be used to study temporal and contextual dynamics of intention–behaviour coupling.
- The paper formulates new research questions and analytic strategies that guide the development of dynamic, context‐sensitive models of health behaviour change and provide a roadmap for future empirical IBG research.

## INTRODUCTION

The intention–behaviour gap (IBG) constitutes a significant and persistent challenge in health psychology, highlighting the complex mechanisms underlying the translation of intentions into behavioural outcomes (Sniehotta et al., 2005). Although theoretical frameworks consistently position intentions as proximal determinants of behaviour (e.g., Theory of Planned Behaviour; Ajzen, 1985), empirical evidence demonstrates substantial discrepancies between individuals' expressed intentions and their subsequent actions (Jekauc et al., 2015; McEachan et al., 2016). This disparity carries considerable implications for public health, as consistent engagement in health‐promoting behaviours, such as physical activity and nutritional practices, plays a crucial role in the prevention of chronic conditions including cardiovascular diseases and diabetes (Dunton, 2018). Furthermore, recognition of the IBG has fundamentally influenced the field of behaviour change by demonstrating that interventions targeting motivational determinants alone are insufficient (Papies, 2017).

Research examining the IBG has predominantly employed between‐person analytical approaches that assess relations of aggregate intentions and behaviours at the group level (Rhodes et al., 2022). While these designs provide valuable information about interindividual differences, they treat intention as a static construct and overlook the substantial fluctuations that occur within individuals across time and context. Traditional cross‐sectional and long‐interval longitudinal studies are therefore limited in their ability to represent the temporal, situational and person‐specific dynamics that shape the enactment of intentions (Knapova et al., 2024; Maher, Behler, et al., 2025).

Several theoretical models have attempted to explain why intentions often fail to lead to behaviour. For example, the Health Action Process Approach distinguishes between motivational and volitional phases and posits that the effects of intention are mediated by action planning and coping planning (Schwarzer, 2016). Temporal Self‐Regulation Theory, by contrast, proposes that the impact of intentions depends on behavioural prepotency and self‐regulatory capacity, and emphasizes the influence of temporally proximal rewards and costs (Hall & Fong, 2007). Although both models offer important insights into mechanisms that facilitate or hinder intention enactment, neither directly conceptualizes the intention–behaviour gap as a dynamic and contextually embedded process that unfolds within individuals over time. Moreover, both frameworks are often operationalized using between‐person designs and trait‐level predictors, which may obscure important within‐person patterns of variability.

Recent works have emphasized that time should be regarded as an integral component of psychological theory rather than a mere control variable (Jekauc et al., 2026; Scholz, 2019). Intensive longitudinal designs such as ecological momentary assessment (EMA) allow researchers to capture the moment‐to‐moment variability in motivation, self‐regulation and contextual influences on behaviour (e.g., Arigo et al., 2022; Haag, Smeddinck, et al., 2025; Knapova et al., 2024; Maes et al., 2022; Maher et al., 2016, 2017; Maher, Behler, et al., 2025; Pickering et al., 2016; Schumacher et al., 2021). However, despite their intuitive appeal, a comprehensive theoretical rationale explaining why such high‐frequency, within‐person designs are critical for advancing IBG research has not yet been systematically articulated.

The Multidimensional Difference Framework (MDF; Jekauc et al., 2025) provides an initial theoretical foundation for conceptualizing the IBG as a phenomenon that varies across person, temporal, contextual and behavioural levels. However, the initial exposition primarily focused on outlining the conceptual dimensions of the framework. The present manuscript extends this framework by elaborating its methodological and analytical implications and by illustrating how these dimensions can be operationalized in empirical research. Integrating theoretical reasoning with advances in intensive longitudinal and person‐centered methodologies, the paper outlines concrete strategies for modelling within‐person processes of intention and behaviour. By refining existing conceptualizations and proposing a dynamic framework, this work aims to contribute to a more precise and empirically grounded understanding of how intentions succeed or fail to translate into action.

## PREVAILING CONCEPTIONS OF THE INTENTION–BEHAVIOUR GAP

The purpose of this section is to clarify how the intention–behaviour gap has been conceptualized within health psychology. Rather than conducting a systematic review, we focus on the two dominant frameworks that have shaped theoretical and empirical discourse on the IBG. These frameworks were selected because they are grounded in meta‐analytic evidence and represent the most influential explanatory traditions in the field. The Explained Variance Approach builds on a series of meta‐analyses that synthesize research using intention‐based predictors of health behaviour, including Hagger et al. (2002), Downs and Hausenblas (2005), McEachan et al. (2011), McDermott et al. (2015) and McEachan et al. (2016). The Action Control Framework is founded by the meta‐analysis of Rhodes and de Bruijn (2013) and was updated by Feil et al. (2023), with a specific focus on physical activity. To our knowledge, these two frameworks represent the most comprehensive and widely cited conceptual models explicitly addressing the intention–behaviour gap in health behaviour research.

### The explained variance approach

The Explained Variance Approach conceptualizes the IBG as the proportion of variance in behaviour that is not accounted for by intention. Using statistical models, such as regression analysis, this approach calculates the residual variance in behaviour after accounting for the predictive power of intention, often expressed as 1 − *R* ^2^, where *R* ^2^ represents the proportion of variance explained by intention. For instance, meta‐analyses have demonstrated that intentions account for approximately 23%–28% of the variance in physical activity behaviour (Hagger et al., 2002; McEachan et al., 2016), suggesting a substantial IBG in which more than 70% of the variance in behaviour remains unexplained by intention (Faries, 2016).

Most studies using this approach adopt between‐person designs, analysing average effects of intention on behaviour across a group (cf., Rhodes, 2024; Rhodes et al., 2022). In these studies, the proportion of (un)explained variance is used as a measure of the IBG's magnitude. Additionally, interactions between intention and other variables are often examined to identify factors that influence the strength of the intention–behaviour relationship. For physical activity research, Rhodes et al. (2022) provided a comprehensive review of the intention–behaviour relationship, primarily focusing on studies employing these between‐person designs. The review identified a number of moderators such as sociodemographic (e.g., employment status) or personality (e.g., conscientiousness) variables that influence the strength of the intention–behaviour relationship. In contrast to this quantitative, variance‐based approach, the Action Control Framework adopts a categorical perspective that focuses on the outcomes of individual intention enactment, offering a complementary but qualitatively distinct lens through which to conceptualize the intention–behaviour gap.

### The action control framework

The action control framework conceptualizes the IBG by classifying individuals based on their success or failure in translating intentions into action. Expanding on Sheeran's (2002) 2 × 2 matrix, this framework categorizes individuals into four distinct groups: successful intenders (those who act on their intentions), unsuccessful intenders (those who fail to act despite having formed an intention), disinclined actors (those who engage in a behaviour without prior intention) and abstainers (those who neither intend to act nor engage in the behaviour). The IBG is operationalized as the proportion of unsuccessful intenders relative to the total number of intenders:

$$\mathrm{IBG}=\frac{\text{Unsuccessful Intenders}}{\text{Successful Intenders}+\text{Unsuccessful Intenders}}$$

For example, Rhodes and de Bruijn (2013) applied this formula to physical activity and determined that 46% of individuals who intended to engage in physical activity failed to follow through, resulting in an IBG of 46% for this behaviour. This framework provides a concrete measure of the IBG, allowing researchers to examine the prevalence of successful intenders within a group of all intenders.

Empirical investigations employing the Action Control Framework typically rely on categorical analyses that compare successful and unsuccessful intenders. Statistical techniques such as logistic regression or discriminant analysis are commonly utilized to identify psychological and contextual factors that differentiate these groups. The framework facilitates the examination of between‐person moderators that influence action control, including self‐regulatory competencies, perceived barriers and external constraints (Rhodes, 2024). Additionally, by classifying individuals into distinct behavioural categories, this approach supports the development of targeted interventions aimed at enhancing action control and increasing the likelihood of behavioural enactment. However, its reliance on a dichotomous classification of success and failure introduces certain limitations, which are discussed in the next sections.

## METHODOLOGICAL CRITIQUE

The dominant conceptualizations of the IBG rely on between‐person research designs, which analyse the average relationship between intentions and behaviour across individuals. Both the Explained Variance Approach and the Action Control Framework are based on statistical methods (e.g., linear and logistic regression or discriminant analyses) that focus on the group level. Although these approaches have yielded important insights into average effects and interindividual differences, their reliance on between‐subject analyses often leads researchers to infer that the relationships observed at the group level also apply at the individual level.

This generalization is problematic because it implicitly assumes ergodicity (Molenaar, 2004). Broadly speaking, ergodicity refers to the condition under which statistical relationships observed across individuals at a single time point are representative of the relationships that occur within an individual over time. More formally, a psychological process is ergodic if its within‐person statistical moments (e.g., means, variances) equal its between‐person moments. Most importantly, this requires the conditions of homogeneity and stationarity. If the ergodic assumptions are met, then it is valid to use group‐level findings to draw inferences about individual‐level processes. However, empirical research in psychology consistently shows that most psychological processes – including those underpinning the IBG – are non‐ergodic (Fisher et al., 2018).

Non‐ergodicity implies that relationships observed at the group level usually do not reflect the processes occurring within individuals over time. For example, across a group, intention and behaviour may show a moderate correlation, suggesting that people with stronger intentions are generally more likely to act. However, this between‐person correlation does not imply that a given individual's weekly variation in intention reliably predicts their own behaviour. An individual may regularly act even when their intention is low (due to habit and contextual facilitators) or fail to act despite high intention (due to external constraints). Without accounting for these person‐specific fluctuations, group‐level findings can obscure the actual dynamics of behaviour regulation. This distinction underscores the necessity of within‐person approaches to accurately understand and model the intention–behaviour relationship.

This methodological contradiction becomes particularly apparent when considering that the IBG is frequently defined as the discrepancy between an individual's intention and their subsequent behaviour – a formulation that is inherently intra‐individual and dynamic. Yet, most empirical studies operationalize the IBG using statistical models that estimate average associations across individuals. Techniques such as linear or logistic regressions do not model individual‐level discrepancies directly but instead produce aggregate estimates. As such, they are incapable of capturing the temporally embedded fluctuations and momentary lapses that characterize how individuals enact or fail to enact intentions in real‐world settings. Treating the IBG as an individual‐level construct while relying on methods that assume homogeneity constitutes a fundamental misalignment between theory and analysis, one that may lead to inaccurate conclusions about the nature, magnitude and causes of the IBG.

## NEGLECT OF THE DYNAMICS OF INTENTION AND BEHAVIOUR

Another major limitation in prevailing IBG research is the insufficient consideration of the temporal and contextual dynamics that characterize intention and behaviour as inherently variable processes. Most studies continue to rely on cross‐sectional or longitudinal designs with extended measurement intervals (Haag, Smeddinck, et al., 2025). This methodological approach overlooks the substantial intra‐individual variability that occurs across short time frames – both in the strength of motivational states and in the context‐dependent content of intentions – leading to a distorted representation of how intentions translate into action in everyday life (Conroy et al., 2011).

Cross‐sectional designs are particularly limited in their ability to capture the temporal unfolding from intention to behaviour. By assessing both constructs at a single time point, these designs preclude examination of temporal precedence and dynamic and contextual change. They offer no insight into how transient shifts in motivation, opportunity or contextual demands impact whether intentions are enacted. As such, they are ill‐suited to investigate a process as temporally contingent as the IBG.

Although longitudinal designs offer some improvement by addressing change over time, those employing extended intervals – such as monthly or biannual assessments – often lack the temporal resolution needed to detect short‐term fluctuations in intention and behaviour. As Mulder et al. (2025) argue, valid inference requires that the temporal resolution of measurement matches the timescale of the psychological process under investigation. For instance, Hamaker (2023) illustrates that when behavioural outcomes referencing a single week are paired with biannual measurements, less than 1% of the relevant behavioural process is captured, severely limiting explanatory power.

Empirical findings from intensive longitudinal studies provide strong evidence against the assumption of temporal stability. Conroy et al. (2013) demonstrated that approximately 50% of the variance in physical activity intentions and over 75% of the variance in daily behaviour resided within individuals. Similar findings have been observed across different populations and measurement intervals. Knapova et al. (2024) found that within‐person variability accounted for 71% of the variance in daily physical activity intentions and 60% in daily step counts. These findings indicate that intention and behaviour are not stable traits but dynamic processes subject to frequent and meaningful shifts.

## INADEQUATE OPERATIONALIZATION

The methodological rigour of IBG research is significantly compromised by inconsistencies in the operationalization of its core constructs: intention and behaviour. For an empirical investigation of the IBG to yield valid and interpretable results, these constructs must be precisely aligned in both content and temporal scope (Ajzen, 1991, p. 185). A widely adopted standard for this alignment is the TACT framework – Target, Action, Context and Time – originally proposed by Ajzen and Fishbein (1977). According to this framework, an intention statement (e.g., “I intend to jog for 30 minutes at the park after work three times a week”) must correspond closely to the behaviour being measured, with consistency across all four TACT elements. However, prevailing research often fails to maintain this alignment, leading to systematic biases in estimating the relationship between intention and behaviour.

A primary issue concerns the conceptual incongruence between the measures of intention and behaviour. Intention is frequently assessed using broad, generalized statements, such as an individual's commitment to engage in regular physical activity. In contrast, behaviour is often quantified through specific metrics, such as the number of minutes spent in moderate‐to‐vigorous physical activity per week (see also Conner & Norman, 2022, for further examples). This mismatch results in a conceptual disconnect wherein intention and behaviour do not reference the same phenomenon with sufficient specificity. From a mathematical and logical perspective, this misalignment introduces measurement error and leads to an underestimation of the strength of the intention–behaviour relationship (Morwitz & Munz, 2021). In order to ensure conceptual validity, intention must be operationalized in a manner that corresponds directly to the subsequent behavioural measurement, maintaining consistency in specificity and definition.

A second critical issue pertains to the temporal and contextual misalignment between intention and behaviour. Intention is often measured with reference to an extended or indefinite future period (e.g., ‘I intend to exercise regularly over the next six months’), whereas behaviour is assessed within a much narrower and specific timeframe (e.g., ‘How many minutes of physical activity did you engage in last week?’). This disparity in temporal reference points introduces a fundamental methodological flaw, as it violates the basic principles of measurement validity (Mulder et al., 2025). The logical and statistical relationship between intention and behaviour is necessarily biased when their temporal frames of reference do not correspond.

Assessing intention–behaviour relations within persons and over shorter timescales offers a direct remedy to these forms of misalignment. Intensive longitudinal approaches such as ecological momentary assessment make it possible to capture intentions that are temporally linked to a specific behavioural opportunity. For example, rather than asking participants whether they intend to ‘attend a fitness course regularly over the next six months’, they can be asked each day from Monday to Friday, ‘Do you intend to attend the fitness course this Friday?’ The actual attendance on Friday can then be recorded as the corresponding behavioural outcome. In this case, both intention and behaviour refer to the same target, action, context and time frame, thereby fulfilling the TACT criteria (Fishbein & Ajzen, 2010). This repeated short‐term measurement allows researchers to observe how daily fluctuations in intention strength predict eventual enactment, reducing conceptual ambiguity and temporal mismatch. A recent study has referred to such micro‐timescale expressions of intention as ‘transitional intentions’, which help bridge the gap between general goal intentions and context‐specific behaviours (Haag, Aulbach, et al., 2025). Consequently, within‐person assessments across short timescales enhance construct validity and yield a more accurate understanding of the processes underlying the intention–behaviour gap (Jekauc et al., 2025).

## CONSEQUENCES AND IMPLICATIONS

Decades of research on the IBG have yielded valuable insights, but dominant approaches remain limited by static assumptions and reliance on between‐person methodologies. As discussed in the previous sections, these models often fail to account for intra‐individual variability, temporal instability and contextual specificity, factors critical for understanding real‐world behaviour regulation. Given these constraints, a reconceptualization of the IBG is warranted.

The following sections outline a multidimensional, dynamic perspective on the IBG. This approach reframes the gap not as a fixed residual or dichotomous outcome, but as a temporally extended, contextually embedded discrepancy between intended and enacted behaviour. Implications of this reconceptualization are discussed with respect to theory, research design, analytic strategy and intervention development.

### Consequences for theory

**FIGURE 1:**
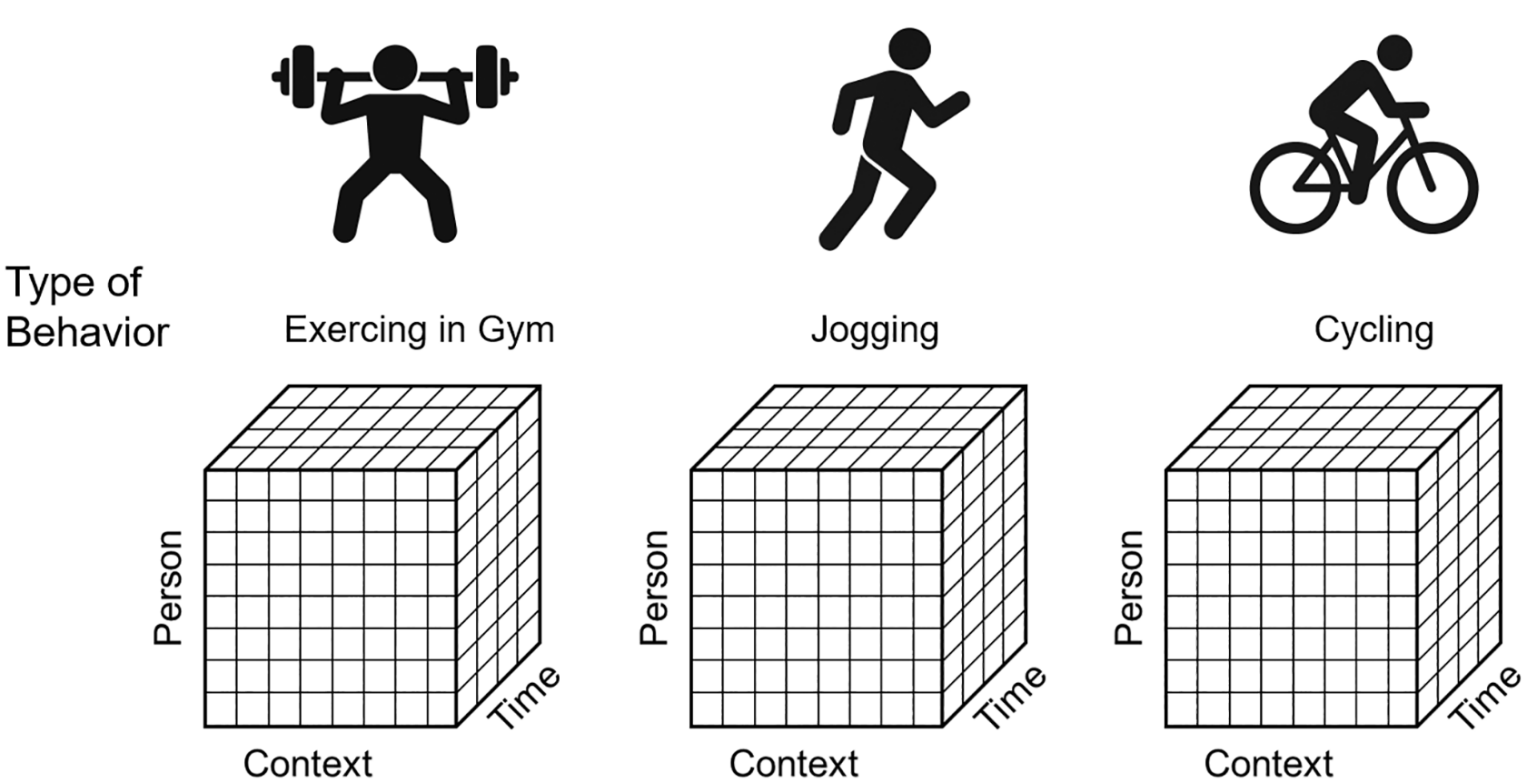
The four dimensions of the intention–behaviour gap: person, time, context and type of behaviour.

In contrast to traditional models that conceptualize the IBG as a static phenomenon or as a residual difference between people, recent theoretical developments propose a new perspective (Jekauc et al., 2026). The Multidimensional Difference Framework advances this shift by redefining the IBG as a dynamic, within‐person process that reconceptualizes the IBG as a multidimensional construct (Jekauc et al., 2025). The MDF framework departs from conventional assumptions that intention and behaviour are linked through fixed or universal mechanisms. Instead, this framework defines the IBG as a temporally extended and contextually embedded discrepancy that exists between intended behaviour and actual enacted behaviour. This reconceptualization emphasizes the dynamic nature of the intention–behaviour relationship and accounts for individual variability in behavioural outcomes across different temporal and situational contexts. This approach shifts the theoretical lens from generalized prediction to individualized explanation, capturing the fluctuating and multifactorial nature of behaviour regulation in everyday life. Central to this reconceptualization is the view that the IBG unfolds across four interrelated dimensions (see Figure 1):

1. Person dimension: Recognizes that the same intention–behaviour discrepancy may have different psychological meanings across individuals. Individual differences in personality, self‐regulatory capacity and motivational orientation shape how intentions are formed and implemented. Empirical evidence from EMA research supports this person‐level variability. Pickering et al. (2016) demonstrated that individuals differ in the extent of their momentary fluctuations in intentions and that greater within‐person variability in intentions is associated with higher daily levels of physical activity. Similarly, Maher, Labban, et al. (2025) found that greater subject‐level variability in daily determinants such as intentions, self‐efficacy and planning predicts higher levels of physical activity over time. These findings indicate that the stability and range of intraindividual fluctuations constitute meaningful person‐specific signatures, directly reflecting the person dimension of the MDF.
2. Temporal dimension: Emphasizes that the IBG is inherently dynamic. Discrepancies may emerge, increase or resolve over time – ranging from minutes to weeks. This temporal sensitivity aligns with the assumptions of Temporal Self‐Regulation Theory (Hall & Fong, 2007), which postulates that the likelihood of behavioural enactment is modulated by both temporal proximity of outcomes and the strength of executive control. Recent EMA evidence also underscores the dynamic nature of the intention–behaviour relationship. Studies by Maher et al. (2016) and Maher and Dunton (2020) revealed that intention–behaviour coupling varies across the day, with stronger associations during morning and evening periods on weekdays and weaker associations on weekends. Such findings illustrate that the temporal dimension of the IBG is not constant but shifts with diurnal and contextual rhythms, supporting the MDF's conceptualization of intention–behaviour trajectories as temporally embedded processes.
3. Contextual dimension: Acknowledges that behavioural enactment is embedded in environmental and situational contexts. The same individual may exhibit differing levels of behavioural concordance depending on situational opportunities, competing demands or social affordances.
4. Behavioural dimension: Proposes that the nature of the behaviour itself – whether it is discrete or continuous, simple or complex – influences the likelihood of intention enactment. Some behaviours (e.g., taking medication) are easier to automate and maintain than others (e.g., regular physical activity). In addition, individuals often hold multiple intentions simultaneously, which may compete for limited attentional or temporal resources. For example, the intention to exercise in the gym may conflict with intentions to jog or cycle (see Figure 1). These competing behavioural goals can exacerbate intention–behaviour discrepancies by introducing internal conflicts and prioritization demands (Sniehotta et al., 2016), further complicating the trajectory of behavioural enactment.

This multidimensional view of the IBG calls for a shift from static, trait‐based models to dynamic, state‐based models that capture within‐person variability and temporal dependencies. Redefining the IBG as a time‐indexed discrepancy trajectory – a dynamic function that maps the evolving difference between intention and behaviour over time – opens the door for dynamic systems modelling and person‐specific prediction approaches. Such models would allow researchers to estimate the likelihood of behavioural enactment based on real‐time fluctuations in psychological and contextual states, thereby offering a more precise and mechanistic understanding of behaviour regulation.

To further formalize the dynamic nature of the IBG, the discrepancy between intention and behaviour can be conceptualized as a time‐indexed trajectory – a function that maps the evolving gap between intended and enacted behaviour across successive time points within as well as between behavioural episodes. This trajectory can assume different shapes: a narrowing gap may reflect successful self‐regulation, emerging habit strength or environmental support, whereas a widening gap could signal intention decay, goal disengagement or increasing contextual constraints. In some cases, the trajectory may oscillate, reflecting fluctuations in motivation, competing goals or situational barriers. The shape of this trajectory – whether stable, volatile, converging or diverging – offers insights into the processes underlying behaviour regulation.

Although this manuscript focuses on health‐promoting behaviours such as physical activity, the MDF is designed to be generalizable across behavioural domains. This includes both approach‐oriented behaviours such as exercise and healthy eating, and avoidance‐oriented behaviours such as smoking cessation or alcohol reduction. For example, Kahveci et al. (2025) recently examined smoking reduction and found that daily fluctuations in intention and craving were closely linked to same‐day smoking behaviour. Their findings show that intention–behaviour discrepancies in avoidance‐oriented behaviour also follow dynamic within‐person patterns. The four dimensions of the framework, the person, temporal, contextual and behavioural levels, are flexible enough to incorporate such domain‐specific mechanisms while still offering a unifying structure for modelling intention–behaviour relations across a wide variety of health behaviours. Moreover, the framework is not limited to understanding behaviour within a single domain but can also guide the analysis of how different behaviours emerge, change and interact across multiple episodes and contexts. It thus provides a conceptual foundation for investigating the development, predictors and situational dependencies of both individual and co‐occurring behaviours.

By advancing a framework that integrates person‐specific dynamics, temporal unfolding, contextual modulation and behavioural variability, the MDF lays the conceptual groundwork for a new generation of IBG research. It encourages researchers to ask not merely whether intentions predict behaviour, but when, for whom, under what circumstances and for what kinds of actions they do so.

### Consequences for research designs

The design implications outlined in this section are directly derived from the four dimensions of the MDF. Understanding intention and behaviour as person‐specific, temporally unfolding, context‐sensitive and behaviour‐dependent requires research designs that can capture fluctuations within individuals over time and across situations. Intensive longitudinal methods such as EMA, daily diaries and repeated assessments across discrete behavioural episodes are particularly well suited to operationalizing these dimensions (Reichert et al., 2020).

A central feature of this approach is the use of designs that enable real‐time or near‐real‐time measurement of intentions, behaviours and relevant contextual variables. EMA and diary methods allow for the collection of high‐frequency data across hours, days and weeks. This directly supports the temporal dimension by enabling researchers to observe how intentions evolve and how they translate into behaviour across time. These methods also support the contextual dimension by capturing environmental cues, affective states and competing demands that may influence the enactment of intentions.

Recent studies have successfully applied such designs to investigate the IBG. For example, Arigo et al. (2022) employed EMA to examine midlife women's physical activity intentions and behaviour, demonstrating substantial within‐person fluctuations and context‐sensitive patterns of intention–behaviour coupling. Similarly, Haag, Smeddinck, et al. (2025) used EMA to show that affective states and environmental context significantly predict day‐to‐day variation in the intention–behaviour link. Knapova et al. (2024) applied daily diary methods and found that within‐person variability accounted for a large portion of the variance in physical activity intentions and behaviours. Maes et al. (2022) extended this work to older adults, showing that momentary shifts in intention and self‐efficacy are strongly predictive of behavioural outcomes. Maher, Behler, et al. (2025) demonstrated that both reflective and reflexive processes dynamically influence behaviour maintenance among older adults, using repeated real‐time assessments. Likewise, Maher and Dunton (2020) provided evidence that within‐day fluctuations in motivational states predict movement‐related behaviours among older adults, underscoring the necessity of aligning measurement timescales with behavioural processes. In addition, Pickering et al. (2016) and Schumacher et al. (2021) emphasized the importance of examining momentary behavioural cognitions, while Maher and Dunton (2020) highlighted intra‐day variations in intention strength shaping physical activity enactments.

To better reflect the dynamic structure of the intention–behaviour gap, future research designs should assess multiple discrete behavioural episodes repeatedly within individuals. Because intentions refer to specific planned actions, it is critical that both intentions and behaviours be measured with reference to specific episodes of opportunity for behavioural enactment. This approach eliminates conceptual ambiguity and satisfies the TACT principle by aligning the target, action, context and time (Ajzen, 1991). It also operationalizes the behavioural and temporal dimensions by capturing the development and execution of intentions over time‐bound, concrete episodes.

In addition, including participant‐defined behavioural episodes or reflective prompts addresses the person dimension by incorporating the individual's own understanding of behaviour and context. Repeated measurement across episodes allows researchers to identify conditions under which intentions are more or less likely to result in behaviour, and to reveal patterns of enactment that may be missed in aggregate‐level analyses. These designs reflect the structure of the MDF and promote a more context‐sensitive and fine‐grained investigation of intention–behaviour processes (Jekauc et al., 2025).

Beyond observational and correlational methods, micro‐randomized trials represent an important innovation for generating causal evidence at short time intervals (Klasnja et al., 2015). In a micro‐randomized trial, participants are repeatedly randomized to either receive or not receive a specific intervention component at multiple decision points over time. This design enables researchers to evaluate how context‐sensitive intervention delivery, such as prompts, motivational messages or action planning cues, influences immediate behavioural outcomes (Qian et al., 2022). Within the framework of the Multidimensional Difference Framework, micro‐randomized trials offer a way to experimentally test how changes in psychological states and environmental conditions affect the likelihood that an intention leads to behaviour. For instance, they can be used to determine whether interventions administered during periods of low intention strength are more successful in promoting behaviour enactment. These trials can provide a flexible and rigorous method for identifying causal mechanisms in moment‐to‐moment intention–behaviour processes and serve as a valuable complement to observational research by supporting the development of context‐aware and adaptive intervention strategies.

### Consequences for analysis strategies

The analysis of the intention–behaviour gap, as conceptualized in the MDF, requires a fundamental methodological shift that recognizes its individual‐specific, temporally dynamic, context‐dependent and behaviour‐related nature. A key challenge for this shift involves the assumption of ergodicity. If researchers intend to generalize from between‐person findings to within‐person processes, explicit tests for ergodicity are necessary. Current methods, such as permutation tests comparing within‐person and between‐person moments (Fisher et al., 2018; Molenaar, 2004), can identify violations of ergodicity but cannot confirm it (Hunter et al., 2024). Future work should incorporate quantitative heterogeneity indices to diagnose the extent of nonergodicity. Emerging metrics, such as the Ergodicity Information Index (Golino et al., 2025), will provide valuable diagnostics to quantify the degree of person‐specificity in data structures. Although we cannot expect ergodicity to hold for most psychological processes, it has been proposed to identify specific conditions or subgroups where ergodicity is tenable (e.g., Adolf & Fried, 2019; Golino et al., 2025; Voelkle et al., 2014). This represents a promising research direction that could help reconcile the analysis of between‐person differences with the study of within‐person change in health behaviour (Voelkle et al., 2018).

Empirical studies increasingly demonstrate that within‐person and between‐person associations in health behaviour research can diverge substantially, underscoring the need to separate these levels in analysis. Wong and Gong (2023), using longitudinal data from more than 7000 adults, found that while subjective health was positively associated with physical activity across individuals, within individuals, declines in health were followed by increases in physical activity, particularly among older adults. These findings highlight the need to use analytic strategies that distinguish within‐person dynamics from between‐person trends, including multilevel modelling, time‐series analysis and person‐specific models (cf. Haag et al., 2023).

Linear mixed models (LLMs) offer a foundational analytic approach for disentangling within‐person and between‐person variability in ecological momentary assessment and similar designs. By estimating both fixed and random effects, these models allow researchers to assess how fluctuations in intention predict behaviour at the within‐person level while accounting for inter‐individual differences in overall behaviour tendencies. This makes LMMs a valuable tool for investigating the person and temporal dimensions of the MDF. For example, LMMs can evaluate whether daily changes in intention strength are linked to daily behavioural outcomes and whether these within‐person effects vary across individuals. They are also useful for modelling cross‐level interactions between‐person‐level characteristics and momentary predictors.

In addition to multilevel approaches, we believe person‐specific time‐series models should become central to IBG research. Methods such as Group Iterative Multiple Model Estimation (GIMME; Gates & Molenaar, 2012) and multi‐vector autoregressive (VAR; Fisher et al., 2022, 2024) allow the estimation of individual‐level VAR networks, while empirically preserving only those structures justified across individuals. These models are critical for theory because they avoid the assumption that all individuals behave in the same way. For example, one person's behaviour might follow a strong affect–intention–action sequence, while another's may be driven more by context or habit. Person‐specific modelling helps uncover these individualized mechanisms, offering richer and more accurate theoretical insights into why and when intentions fail to translate into action.

When measurement intervals vary, continuous‐time models offer a powerful data analytic approach by reconstructing how psychological processes evolve continuously, even when measurements are taken at irregular intervals (Oud & Jansen, 2000; Van Montfort et al., 2018; Voelkle et al., 2012). This is particularly useful in real‐world behavioural research, where people's experiences and actions unfold on different time scales (e.g., hours, days or weeks), and where capturing exact timing is crucial for identifying when intentions strengthen or dissipate.

Finally, the methodological framework proposed by Jekauc et al. (2025) complements these dynamic approaches by offering a practical operationalization of the IBG as a multidimensional difference. Their use of within‐person stability metrics, such as the mean square of successive differences (MSSD; von Neumann et al., 1941), captures temporal fluctuations in intentions and behaviours. However, while this approach provides a valuable descriptive index of stability, it does not substitute for explicit dynamic causal modelling, which remains crucial for understanding the mechanisms linking intention and behaviour over time.

### New research questions in health behaviour research

The Multidimensional Difference Framework not only provides a conceptual foundation for understanding the IBG but also generates a new set of guiding research questions for health behaviour science. These questions concern the temporal evolution of intentional discrepancies and the dynamic interplay between intentions, contextual influences and behavioural enactment. Traditional models of the IBG have primarily focused on explaining why intentions often fail to predict behaviour at the group level. In contrast, the MDF encourages a shift towards investigating how intention–behaviour discrepancies arise, stabilize and change within individuals across time and situations. This perspective redirects attention from static associations to dynamic processes, emphasizing that the IBG is not a fixed quantity but a temporally unfolding trajectory that reflects momentary motivational states and situational affordances.

A central question emerging from this perspective is how intentional discrepancies develop and transform over time within a person (Jekauc et al., 2026). The MDF proposes that the size of the IBG depends critically on temporal distance and situational context. For example, the correspondence between intention and behaviour may be high when both are assessed within a short time interval but may diminish as the delay between intention formation and behavioural opportunity increases. Understanding how the predictive power of intentions decays or stabilizes over time can reveal the temporal boundaries of intention‐based regulation and help to identify the time windows in which interventions are most effective (Jekauc et al., 2025). Intensive longitudinal methods, such as ecological momentary assessment (Reichert et al., 2020), are particularly suited to examine these short‐term fluctuations and to trace the micro‐dynamics through which intentions are formed, maintained and enacted in daily life (Maher, Labban, et al., 2025).

Equally important is the examination of intraindividual patterns of the IBG across multiple behavioural episodes. From a dynamic perspective, each episode of intention formation and enactment can be seen as a realization of an ongoing self‐regulatory process (Jekauc et al., 2025). By modelling these sequences over time, researchers can explore whether some individuals show consistent patterns of success or failure in implementing their intentions, whether these patterns are stable or variable, and how they are shaped by motivational, affective or contextual factors. Such analyses allow for identifying person‐specific trajectories that may characterize adaptive or maladaptive forms of behavioural regulation.

Another key direction concerns the micro‐temporal processes that precede a planned behaviour. Before an action occurs, intentions may fluctuate in strength and salience as individuals weigh competing goals, evaluate their resources or experience changes in affect and context (Conroy et al., 2011). Capturing these fine‐grained variations can clarify how momentary (or transitional) intention dynamics influence the probability of enactment at a specific point in time (Haag, Smeddinck, et al., 2025; Maher et al., 2016). For example, assessing intentions repeatedly in the days leading up to a fitness class could reveal whether stable or strengthening intentions predict actual attendance more reliably than variable or weakening ones. These insights can inform the design of temporally sensitive interventions that aim to stabilize or reinforce intentions during critical pre‐action periods.

Finally, the MDF raises the broader question of which factors contribute to the stabilization or alteration of intentions over time. Intentions may become more consistent when they are supported by habits, routines or contextual cues, but they may also weaken when individuals face competing demands or changes in their environment (Haag, Smeddinck, et al., 2025). Investigating the determinants of intention stability and change, including anticipated affective experiences, habit strength, self‐control and contextual affordances, can clarify the mechanisms that sustain or erode motivational commitment. Addressing these questions will help to connect motivational and self‐regulatory theories and to provide a more integrated understanding of how psychological and situational forces jointly influence behaviour.

Together, these lines of inquiry exemplify how the MDF extends the theoretical and methodological agenda of health behaviour research. They transform the study of the intention–behaviour gap from a static examination of mean‐level discrepancies into a dynamic investigation of temporal trajectories, intraindividual variability and contextual modulation. By combining intensive longitudinal designs with advanced analytic techniques, such as time‐varying effect modelling, dynamic structural equation modelling or person‐specific time‐series analysis, researchers can map how intentions and behaviours interact within the temporal flow of everyday life. Such an approach opens new pathways for identifying when and why intentions succeed or fail, ultimately enabling the development of adaptive, just‐in‐time interventions that operate on the same temporal scale as the behaviours they aim to change.

### Practical implications for intervention

Addressing the IBG requires the development of interventions that account for the dynamic, context‐dependent nature of behavioural enactment (Alicia et al., 2019). Traditional intervention strategies often assume a stable relationship between intention and behaviour, failing to consider fluctuations in motivation, situational constraints and self‐regulatory processes (Jones et al., 2025). To enhance the effectiveness of interventions, approaches must be tailored to individual differences, environmental influences and temporal variability in intention and action (Hall & Fong, 2007; Wang & Miller, 2020).

In the motivational phase, forming strong, self‐concordant intentions is essential. Motivational interviewing (MI) is an evidence‐based method to enhance intrinsic motivation, resolve ambivalence and increase readiness for change (Rubak et al., 2005). Although MI may not consistently outperform other techniques in isolation, it is a valuable component of multi‐phase interventions, particularly when combined with planning and habit‐forming strategies (Zhu et al., 2024). Interventions that focus on internalized goal setting rather than external pressures tend to result in more enduring behaviour change.

During the volitional phase, implementation intentions have shown to be one of the most effective tools for bridging the intention–behaviour gap (Bélanger‐Gravel et al., 2013). These plans specify critical situational cues and link them to predetermined responses (e.g., ‘If I feel tired after work, then I will still go to the gym for 20 min’). By mentally rehearsing such contingencies, individuals enhance the accessibility of relevant opportunities or obstacles and automate their responses when these situations arise. A meta‐analysis of 94 studies demonstrated that forming if‐then plans yields medium‐to‐large improvements in goal attainment (*d* = .65) compared to merely forming goal intentions (Gollwitzer & Sheeran, 2006). Implementation intentions have been shown to facilitate action initiation, maintenance and closure, as well as overcoming procrastination, distractions, temptations and emotional barriers (Haag, Aulbach, et al., 2025; Sheeran & Webb, 2016). These plans operate by forging strong associative links between situational cues and goal‐directed responses, thereby allowing for bottom‐up, cue‐driven action control akin to habitual behaviour (Gollwitzer, 1999; Wood & Neal, 2007).

Given that behavioural enactment is influenced by momentary fluctuations in psychological and contextual states, real‐time adaptive strategies are needed. Just‐in‐Time Adaptive Interventions (JITAIs) deliver support at critical moments, e.g., when a person is most receptive or vulnerable, based on EMA data. These interventions can prompt action, reinforce motivation or mitigate competing demands precisely when behavioural decisions are made (Fiedler et al., 2024; Hardeman et al., 2019). JITAIs are particularly effective in addressing acute lapses in self‐control or motivation that disrupt the intention–behaviour link (Klasnja et al., 2018).

While self‐regulatory strategies are essential for initiating behaviour, long‐term maintenance is more reliably achieved through habit formation (Strobach et al., 2020). Repetitive enactment in consistent contexts fosters automaticity, reducing reliance on conscious intention (Lally & Gardner, 2013). Recent meta‐analytic evidence confirms that habit‐based interventions significantly enhance physical activity and habit strength (Ma et al., 2023). Effective habit‐building techniques include context cueing (e.g., linking activity to existing routines), consistency in behavioural context and reinforcing intrinsic rewards (Hagger, 2019). These strategies reduce cognitive load and protect against motivational lapses, which are frequent disruptors of long‐term behaviour change.

## CONCLUSION

The present paper critically examined the prevailing conceptualizations and methodological approaches to the IBG, identifying key limitations that constrain its theoretical and empirical understanding. These limitations highlight the need for a broader perspective that accounts for the dynamic and context‐dependent nature of the IBG. A multidimensional perspective that incorporates frequent measurement and context‐sensitive analyses can bridge this gap by acknowledging the interplay between motivational, environmental and self‐regulatory factors. Furthermore, refining the core constructs of intention and behaviour – ensuring conceptual and temporal alignment – will enhance measurement congruency, validity and theoretical precision.

Recognizing intra‐individual variability and contextual influences is deemed essential for methodologically bridging the gap between research and practice. By shifting from static, group‐level correlations to more nuanced, process‐oriented models, IBG research can move towards a deeper, more actionable understanding of how intentions evolve into behaviour. Future studies should embrace flexible designs, intensive longitudinal methods and advanced analytical techniques to more accurately capture the complexities of human behaviour. This approach will not only refine theoretical models but also improve the effectiveness of interventions aimed at promoting sustained behaviour change in real‐world settings.

## AUTHOR CONTRIBUTIONS

**Darko Jekauc:** Conceptualization; writing – original draft; writing – review and editing. **Manuel C. Voelkle:** Conceptualization; methodology; writing – review and editing. **Falko F. Sniehotta:** Visualization; writing – review and editing. **Claudio R. Nigg:** Writing – review and editing.

## Data Availability

No primary data were generated or analysed in the preparation of this conceptual manuscript. Therefore, no data are available for sharing.
